# Retinol Dehydrogenases Regulate Vitamin A Metabolism for Visual Function

**DOI:** 10.3390/nu8110746

**Published:** 2016-11-22

**Authors:** Bhubanananda Sahu, Akiko Maeda

**Affiliations:** 1 Department of Ophthalmology and Visual Sciences, School of Medicine, Case Western Reserve University, Cleveland, OH 44106-4965, USA; bxs342@case.edu; 2 Department of Pharmacology, Case Western Reserve University, Cleveland, OH 44106-4965, USA

**Keywords:** visual cycle, retina, retinal pigmented epithelium, vitamin A, All-*trans*-retinol, retinol dehydrogenase, fundus albipunctatus

## Abstract

The visual system produces visual chromophore, 11-*cis*-retinal from dietary vitamin A, all-*trans*-retinol making this vitamin essential for retinal health and function. These metabolic events are mediated by a sequential biochemical process called the visual cycle. Retinol dehydrogenases (RDHs) are responsible for two reactions in the visual cycle performed in retinal pigmented epithelial (RPE) cells, photoreceptor cells and Müller cells in the retina. RDHs in the RPE function as 11-*cis*-RDHs, which oxidize 11-*cis*-retinol to 11-*cis*-retinal in vivo. RDHs in rod photoreceptor cells in the retina work as all-*trans*-RDHs, which reduce all-*trans*-retinal to all-*trans*-retinol. Dysfunction of RDHs can cause inherited retinal diseases in humans. To facilitate further understanding of human diseases, mouse models of RDHs-related diseases have been carefully examined and have revealed the physiological contribution of specific RDHs to visual cycle function and overall retinal health. Herein we describe the function of RDHs in the RPE and the retina, particularly in rod photoreceptor cells, their regulatory properties for retinoid homeostasis and future therapeutic strategy for treatment of retinal diseases.

## 1. Introduction

Vitamin A and its derivatives play important roles not only in development of the eye but also in day-to-day visual function. The most significant role of vitamin A in vision is to regenerate the visual chromophore of rhodopsin for receiving light. Light perception in vertebrates is initiated by activation of rhodopsin, which leads to a cascade reaction starting with stimulation of the G protein called phototransduction in the photoreceptor outer segments [1]. To make rhodopsin light sensitive, covalent linkage between a vitamin A derivative 11-*cis*-retinal and opsin is essential. Photoisomerization of 11-*cis*-retinal to all-*trans*-retinal causes conformational changes in the opsin molecule that enable it to stimulate transducin. Continuous vision depends on recycling of the photoproduct all-*trans*-retinal back to visual chromophore 11-*cis*-retinal. This process is enabled by the visual (retinoid) cycle, a series of biochemical reactions in photoreceptor, adjacent RPE and Müller cells. Production of visual chromophore to bind rhodopsin in the rod photoreceptor is largely understood and is executed by RPE cells, which is called the rod visual cycle or canonical visual cycle [2]. Accumulating evidence supports the notion that rapid production of visual chromophore in the cone photoreceptor is performed by mainly Müller cells, which is called the cone visual cycle [3]. Retinoid dehydrogenases (RDHs) play important roles in the rod and cone visual cycle. In this review, the rod visual cycle, which biochemistry has been well studied, is mainly discussed as the visual cycle. Impairments in the visual cycle can cause retinal degeneration and mutations in RDHs can be detected in human retinal diseases.

## 2. The Visual Cycle

The principal reactions of the visual cycle were delineated by George Wald in the 1940s. He discovered 11-*cis*-retinal which is bound to opsin by Schiff base formation to produce light sensitive rhodopsin [4,5,6]. He also proposed that, after exposed to light, all-*trans*-retinal was dissociated from rhodopsin. These important discoveries brought him the Nobel Prize in 1967 in Physiology and Medicine [7]. 

**Figure 1:**
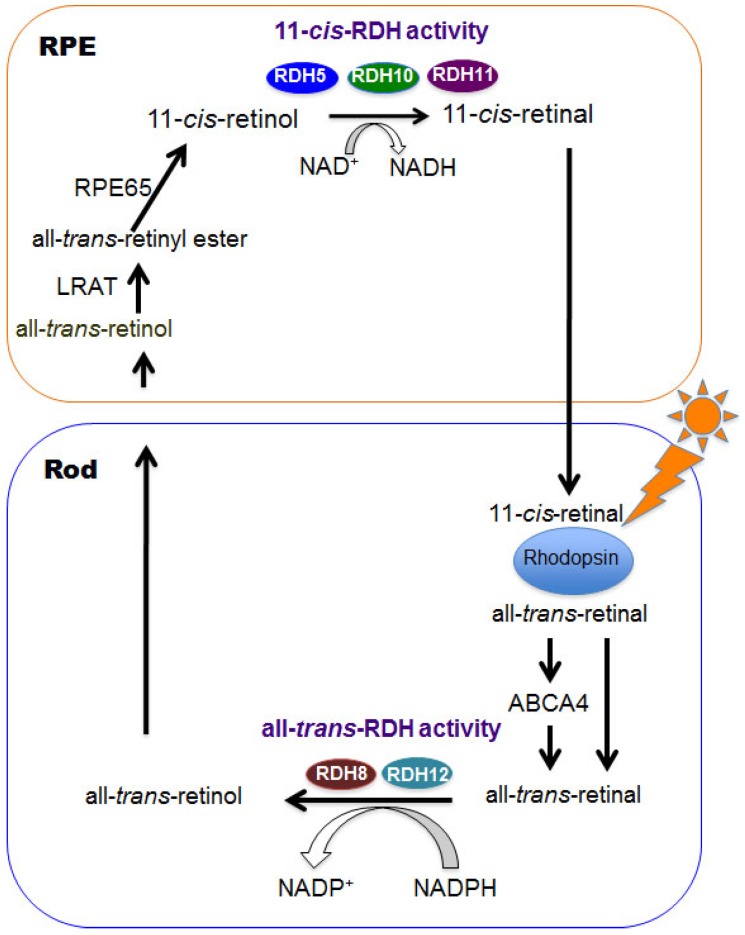
Rod visual cycle is shown. Visual chromophores, vitamin A derivatives, are regenerated via this cycle. RDHs play an important role for this process. RPE, retinal pigmented epithelium; RDH, retinol dehydrogenase; LRAT, lecithin retinol acyltransferase; RPE65, retinal pigmented epithelium-specific 65-kDa; ABCA4, ATP-binding cassette transporter 4.

Rhodopsin activation is initiated by photoisomerization of 11-*cis*-retinal to all-*trans*-retinal in 50 femtoseconds [8,9], and photo signals are converted to electrical signals which are transduced to the visual cortex of the brain. The visual cycle is a series of biochemical reactions to regenerate 11-*cis*-retinal from photoisomerized chromophore all-*trans*-retinal (Figure 1). All-*trans*-retinal is reduced to all-*trans*-retinol by all-*trans*-RDHs including RDH8 [10] and RDH12 [11]. A fraction of all-*trans*-retinal that enters the luminal space of the disc in photoreceptor outer segments is flipped by the ATP-binding cassette transporter 4 (ABCA4) to the cytoplasmic side where it can be accessed as a substrate by all-*trans*-RDHs [2]. All-*trans*-retinol is transported to the intra photoreceptor space where it is chaperoned by intra photoreceptor binding protein (IRBP), which carry it to the RPE. In the RPE, all-*trans*-retinol further binds to cellular retinol binding protein 1 (CRBP1) and is esterified by lecithin retinol acyltransferase (LRAT) [2]. Retinal pigmented epithelium 65 (RPE65) catalyzes the reaction from all-*trans*-retinyl ester to 11-*cis*-retinol. The final process of the cycle from 11-*cis*-retinol to 11-*cis*-retinal is conducted by 11-*cis*-RDHs including RDH5, RDH10 and RDH11. The above biochemical process is the canonical visual cycle and is also called the rod visual cycle. 

There is a cone photoreceptor specific visual cycle for visual function at daytime. In this cone, visual cycle all-*trans*-retinal is reduced to all-*trans*-retinol by RDH8, which is transported to Müller cells where all-*trans*-retinol is isomerized to 11-*cis*-retinol by the recently discovered DES1 enzyme [12]. Finally, 11-*cis*-retinol is carried away to cone cells where it is oxidized to 11-*cis*-retinal by some unidentified retinol dehydrogenases [13]. Recently, the cone dominant retina of carp displays efficient oxidation of 11-*cis*-retinol by dehydrogenases in absence of NADP^+^ or NAD^+^. Instead simultaneous reduction of all-*trans*-retinal to all-*trans*-retinol is needed which is called retinal-retinol redox (AL-OL)-coupling reaction. AL-OL coupling reaction supplies 11-*cis*-retinal at the 240 times higher rate in cone photoreceptor cells as compared to rod visual cycle [14]. 

## 3. Short Chain Dehydrogenases/Reductases (SDRs)

RDHs belong to the family of short-chain dehydrogenases/reductases (SDRs) protein with a polypeptide of approximately 250 to 350 amino acid sharing 20%–30% identity in pair wise comparison indicating distance duplication [15]. The SDR family proteins have a conserved structure of Rossmann-fold comprised of 6-7 β-strands, which are flanked by 3-4 α-helices from each side. The strand topology is 3-2-1-4-5-6-7 with a long cross over binding site between strand 3 and 4 making a binding site for nicotinamide [16]. The SDR super family has more than 120,000 members, which are distributed in 464 families [17] (http://sdr-enzymes.org). The SDR family protein has two characteristically conserved domains [1] *N*-terminal glycine rich motif (GXXXGXG) for binding to nucleotide and [2] *C*-terminal catalytic tetrad N-S-Y-K, which constitutes the active site [18]. In contrast to medium chain dehydrogenase/reductases (MDRs) including alcohol dehydrogenases which require Zn for its catalytic activity, the SDR family protein does not require any metal for its catalytic activity [19]. A wide variety of molecules such as steroids, fatty acid and retinoids are substrates for the SDR family of enzymes [17].

## 4. Retinol Dehydrogenases (RDHs) in the RPE

RDHs in the RPE in vivo catalyze the final oxidation step in the visual cycle namely conversion of 11-*cis*-retinol to 11-*cis*-retinal, the visual chromophore of mammalian visual pigments. These RDHs are also named as 11-*cis-*RDHs. RDH5, RDH10 and RDH11 have been identified in the RPE as 11-*cis*-RDHs.

### 4.1. RDH5

In 1995, Simon et al. isolated RDH5 as a 32-kDa membrane-associated protein (p32) consisting of 318 amino acids, which forms a complex with RPE65 [20]. 

#### 4.1.1. Expression and Localization

**Figure 2:**
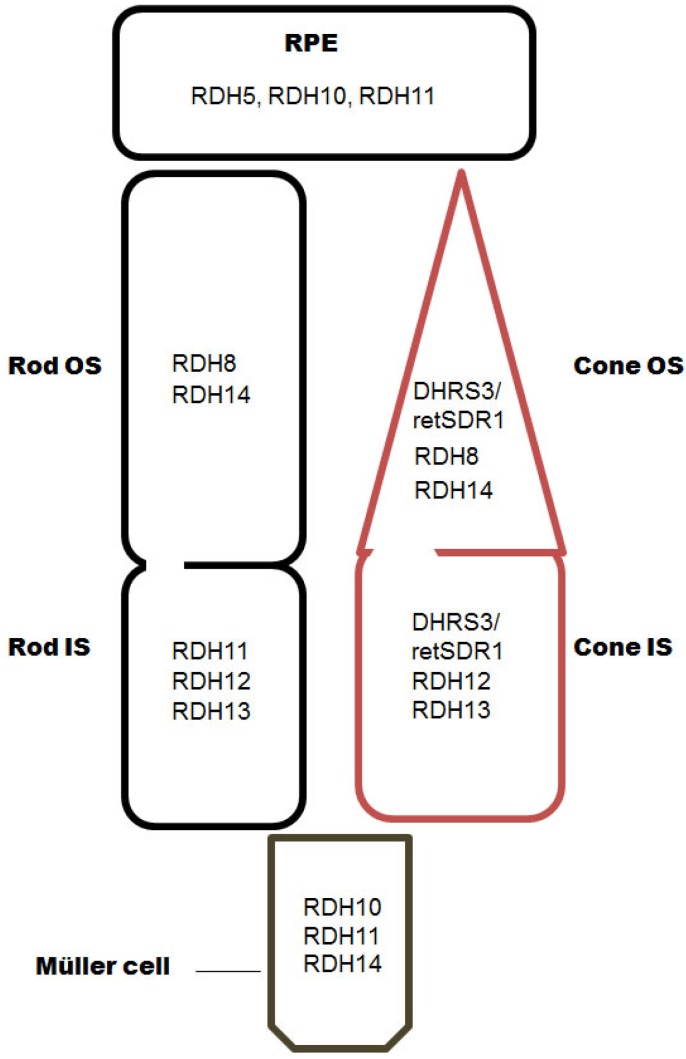
Localization of RDHs in the RPE and the retina. OS, outer segments; IS, inner segments.

The human *RDH5* gene locates on chromosome 12 at 12q13-q14 and chromosome 10 in mice, and is expressed predominantly in the RPE (Figure 2). Apart from the RPE, RDH5 also expresses in the liver (100–500 fold less as compared to the RPE), mammary gland, colon, thymus, small intestine, kidney, bladder, pancreas and spleen [21]. RDH5 anchors to RPE microsomes by its *N*- and *C*-terminal transmembrane segment and the catalytic domain is located towards the luminal side of the microsome [22]. The eight amino acids at *C*-terminal tail of RDH5 is proposed to be crucial for activity and mutations within or close to this tail identified in patients with Fundus albipunctatus (FA) [23,24,25]. Therefore, this tail proposed to have putative role in interacting with protein present in the cytoplasmic side of microsomes. RDH5 is found as a dimer or tetramer protein. Additionally, it is postulated that murine Rdh5 displays a cytosolic topology with *N*-terminal anchoring to the membrane [26,27]. RDH5 has been reported to localize in the endoplasmic reticulum (ER) [22,28].

#### 4.1.2. Biochemical Properties

RDH5 displays NAD^+^ preference and has highest specificity towards 11-*cis*-retinal followed by 13-*cis*-retinal and 9-*cis*-retinal [20,29,30]. It also recognizes steroids (5α-androstan-3α, androsterone) as its substrates [21]. 

#### 4.1.3. Animal Studies

*Rdh5*^−/−^ mice failed to mimic the phenotype observed in humans with RDH5 mutations [29]. Dark adaption is normal in *Rdh5*^−/−^ mice and no white spot are visible in their fundus. Under intensive light conditions, *Rdh5*^−/−^ mice exhibit a delay in dark adaption. One of the predominant features of *Rdh5*^−/−^ phenotype is accumulation of 11-*cis* and 13-*cis*-retinyl esters [29,31,32,33]. The residual dehydrogenase in *Rdh5*^−/−^ mice is responsible for production of 11-*cis*-retinal and 9-*cis*-retinal but not 13-*cis*-retinal [30]. Lack of 13-*cis*-RDH activities is a reason for accumulation of 13-*cis*-retinyl esters. The accumulation of 13-*cis*-retinyl esters could be related to white dots in human retinas due to RDH5 mutations [2].

#### 4.1.4. Disease

Mutations in RDH5 can cause autosomal recessive Fundus albipunctatus (FA), which is characterized by numerous white dots in fundus [34]. FA was initially reported as congenital stationary night blindness, but more recent studies revealed that 30% of these patients develop cone dystrophy in their 4th decade of life [35,36,37,38]. 

### 4.2. RDH11

*RDH11* gene was initially known as prostate short-chain dehydrogenase 1 (PSDR1) since it was first discovered in a prostate cancer cell line after exposure to androgen [39]. Later, Rdh11 was identified to be up-regulated in adipose tissues (brown and white) and the liver in transgenic mice with over expressed sterol regulatory element-binding protein-2 (SREBP-2). SREBPs are transcription factors that bind to the sterol regulatory element DNA sequence and facilitate cholesterol and fatty acid biosynthesis [40]. RDH11/PSDR1 is also recognized as retinal reductase 1 (RalR1) [41] and short-chain aldehyde reductase (SCALD) [42].

#### 4.2.1. Expression and Localization

Human *RDH11* gene locates on chromosome 14 at 14q24.1 and exhibits 85% identity to murine *Rdh11* that locates on chromosome 12. In humans, RDH11 is expressed in wide varieties of tissues such as the kidney, pancreas, liver, testis and prostate [43]. Immunohistochemistry assay revealed a signal of RDH11 expression in the RPE in monkey and bovine eyes, whereas a faint signal was found in the rod photoreceptor inner segment and Müller cells [43]. More recent studies with mice found Rdh11 expression in the rod photoreceptor inner segment [31,44] (Figure 2). RDH11 locates in microsomes with the help of the *N*-terminal hydrophobic segment [41]. The *N*-terminal domain is anchored to the membrane (residue 2–22 reference to human RalR1 polypeptides) and the rest of the polypeptide faces to the cytosolic side of the membrane [45]. RDH11 has also been reported to localize to the ER [41]. 

#### 4.2.2. Biochemical Properties

Although RDH11 can catalyze both oxidation and reduction depending upon cofactors and substrates [43], NADPH is a preferred cofactor for RDH11. RDH11 can catalyze a reduction of retinal ~50-fold more efficiently than oxidation of retinol in vitro [41]. RDH11 also has dual substrate specificity towards both *cis*- and *trans*-retinoids such as all-*trans*-retinal, 9-*cis*-retinal and 11-*cis*-retinal. 

#### 4.2.3. Animal Studies

RDH5 is responsible for most of the 11-*cis*-RDH activity in the RPE, but formation of 11-*cis*-retinal in *Rdh5*^−/−^ mice suggests that another enzyme(s) is present. One of these enzymes is RDH11. Although *Rdh11*^−/−^ mice failed to show a significant dysfunctional retinal phenotype, 73% more *cis*-retinyl esters were detected in *Rdh5*^−/−^*Rdh11*^−/−^ mice than in *Rdh5*^−/−^ mice after intense light illumination. *Rdh5*^−/−^*Rdh11*^−/−^ mice displayed normal ERG responses under dark- and light-adapted conditions, but an enhanced delay in dark adaptation was observed as compared with *Rdh5*^−/−^ mice. These studies with mice revealed that RDH11 has a complementary role in 11-*cis-*retinal regeneration along with RDH5 [31].

#### 4.2.4. Disease

A compound heterozygous nonsense mutation c.C199T:p.R67 and c.c322T:p.R108 in RDH11 has been reported in a syndromic retinitis pigmentosa [46]. The syndromic features in the patient include facial dysmorphologies, psychomotor developmental delays since childhood, learning disability and short stature. 

### 4.3. RDH10

RDH10 was cloned from the retina in humans, bovines and mice [47]. 

#### 4.3.1. Expression and Localization

RDH10 gene encodes a protein of 341 amino acids in humans and mice, and locates on chromosome 8 at 8q21.11 and chromosome 1, respectively. Human RDH10 has high sequence identity to bovine and mouse. RDH10 predominantly expresses in the microsomal fraction of the RPE along with the Müller cells, kidney, liver, small intestine, placenta, lung, heart and skeletal muscle (Figure 2). RDH10 has two hydrophobic sites (2–23 and 293–329), which are proposed to anchor the microsomal membrane of the RPE [48]. *Rdh10*^−/−^ mice show embryonic lethality, and therefore RDH10 is an essential enzyme for synthesis of embryonic retinoic acid [49]. RDH10 is demonstrated to be co-localized with RPE65 and CRALBP in the RPE indicating involvement in the visual cycle [50]. It localizes to the ER [51].

#### 4.3.2. Biochemical Properties

A preferred cofactor for RDH10 is NAD^+^ and the sequence analysis revealed that aspartic acid at position 67 in the characteristic βαβ region would favor the NAD^+^ binding [32,52]. The substrate specificity of RDH10 is as follows all-*trans*-retinol > 9-*cis*-retinol >11-*cis*-retinol [32].

#### 4.3.3. Animal Studies

RPE-specific deletion of Rdh10 (rtTA-Cre^+/−^*Rdh10*^flox/flox^, *cRdh10*KO) does not show any retinal abnormalities under room light conditions. However, with an intense light condition, *cRdh10*KO mice display a delayed dark adaption and a delay in 11-*cis*-retinal regeneration. Double knockout of *Rdh10* and *Rdh5* (*Rdh5*^−/−^*cRdh10*KO) also has normal retinal morphology, which is an indication of redundancy of retinol dehydrogenase in the RPE [32]. 

#### 4.3.4. Disease

There is no report of RDH10 involvement in human retinal diseases.

## 5. Retinol Dehydrogenases (RDHs) in the Retina

RDHs that are present in the retina are also called all-*trans*-RDHs. These RDHs reduce all-*trans*-retinal to all-*trans*-retinol in presence of NADPH as a cofactor. RDH8, RDH12–14 and retSDR1 have been identified in the retina with all-*trans*-RDH activity as described below.

### 5.1. RDH8

RDH8 (also known as photoreceptor RDH, prRDH) was first identified by Rattner and colleagues from the bovine retina using cDNA subtraction, normalization and high throughput sequencing [53]. Bovine and human RDH8 are highly homologous and exhibits 48% identity with 17-hydroxysteroid dehydrogenase type 1.

#### 5.1.1. Expression and Localization

Human *RDH8* gene encodes a polypeptide of 331 amino acids and presents on chromosome 19 at 19p13.2 whereas mouse *Rdh8* encodes 317 amino acids with location on chromosome 9. RDH8 expression is limited to the outer segments of cone and rod photoreceptors [53] (Figure 2). RDH8 is an enzyme anchored to the outer segment of the photoreceptor with its *C*-terminal 16 amino acids. This region contains palmitoylation on three potential cysteines, which are conserved among different species [15]. 

#### 5.1.2. Biochemical Properties

RDH8 used NADPH and all-*trans*-retinal as its preferred cofactor and substrate, respectively [53,54].

#### 5.1.3. Animal Studies

*Rdh8*^−/−^ mice display normal retinal morphology and retinal function under room lighting conditions [10]. However, after light exposure, accumulation of all-*trans*-retinal and delayed dark adaption was observed [10]. Delayed clearance of all-*trans*-retinal results in increased amounts of di-retinoid-pyridinium-ethanolamine (A2E) in aged mice. A2E is biosynthesized with two all-*trans*-retinal molecules and one phosphatidylethanolamine, which is abundant in the retina [55]. In retinal diseases including Stargardt disease and age-related macular degeneration (AMD), accumulation of autofluroscence granule called lipofuscin is observed where A2E is the major fluorophore [56]. A2E oxidation products could play a crucial role in activation of complementation factors and inflammation in retinal disease [57,58,59]. In vivo, clearance of all-*trans*-retinal is conducted by RDH8 together with ATP-binding cassette transporter 4 (ABCA4). ABCA4 flips all-*trans*-retinal which is released into the inner-leaflets of discs to the cytosolic lumens where RDH8 exists [60]. In contrast to normal retinal morphology in *Rdh8*^−/−^ mice, *Rdh8*^−/−^*Abca4*^−/−^ mice display progressive retinal degeneration [61,62,63] and *Rdh8*^−/−^*Abca4*^−/−^ mice serve as a useful animal model to study Stargardt disease and AMD. 

#### 5.1.4. Disease

There is no disease association found in human retinal diseases. Mutations of RDH8 were found in myopia cases [64]. 

### 5.2. RDH12

Haeseleer et al. identified RDH12 (accession number AK054835) together with RDH13 (accession number BE736147) and RDH14 (accession number AF237952). RDH12 expression was found in the inner segment of the photoreceptor in monkeys and mice [43]. 

#### 5.2.1. Expression and Localization

*RDH12* gene of humans encodes 316 amino acids and locates on chromosome 14 at 14q24.1 whereas mouse *Rdh12* locates on chromosome 12 encoding 316 amino acids. RDH12 expresses in the inner segment of rod and cone photoreceptors [65,66] (Figure 2). RDH12 expression was also detected in the kidney, pancreas, liver, prostrate, testis and brain [67]. RDH12 has single α-helix spanning in the membrane and the catalytic domain is present in the cytosol [15]. Subcellular localization of RDH12 is the ER [51].

#### 5.2.2. Biochemical Properties 

RDH12 is a NADPH-dependent reductase and has maximum activity with 9-*cis* and all-*trans*-retinal. It also catalyzes medium-chain aldehydes as its substrates [43].

#### 5.2.3. Animal Studies

*Rdh12*^−/−^ mice fail to display any retinal pathology under room lighting conditions (~50 lux) and do not recapitulate the pathology observed in humans [65,66]. As compared with *Rdh8*^−/−^ mice, delay in all-*trans*-retinal clearance and a slow dark adaption are milder in *Rdh12*^−/−^ mice. *Rdh12*^−/−^ mice display more susceptible to light induced retinal degeneration as compared to WT mice [66,68]. A study from *Rdh8*^−/−^, *Rdh12*^−/−^ and *Rdh8*^−/−^*Rdh12*^−/−^ mice revealed that Rdh8 and Rdh12 impart ~70% and ~30% of all*-trans*-RDH activity in vivo respectively [69]. *Rdh8*^−/−^*Rdh12*^−/−^ mice display mild retinal abnormality by six months of age though 98% of all-*trans*-RDH activity is abolished. Age-related accumulation of A2E was also observed in these three lines of animals, and A2E amounts were correlated with in vivo clearance rates of all-*trans*-retinal after light illumination.

#### 5.2.4. Disease

Mutations of RDH12 can cause an autosomal recessive childhood onset of retinal dystrophy termed as Leber Congenital Amaurosis (LCA) [11,70,71]. Until now, 76 mutations have been reported by HGMD professional where most of mutations are reported as autosomal recessive traits [72]. Patient with LCA exhibit visual impairment with attenuated rod and cone function in early life with macular atrophy, nystagmus, sluggish or absent papillary response, photophobia and night blindness [73,74].

### 5.3. RDH13

Haeseleer et al. identified RDH13 in the retina together with RDH12 and RDH14 [43].

#### 5.3.1. Expression and Localization

Human *RDH13* encodes 331 amino acids and locates on chromosome 19 at 19q13.42. Mouse *Rdh13* encodes 334 amino acids and locates on chromosome 7. Human RDH13 shares 83% protein identity to the mouse counterpart. RDH13 expresses in the eye, pancreas, placenta and lung. Immunohistochemistry revealed RDH13 expression in the inner segment of rod and cone photoreceptors in humans, monkeys and mice (Figure 2). RDH13 shares greatest sequence similarities with RDH11, RDH12 and RDH14, which are integral membrane proteins of the ER. RDH13 localizes to the outer side of the inner mitochondrial membrane [75]. Sub-mitochondrial localization analysis revealed that RDH13 is not an integral but a peripheral protein anchored to the *N*-terminal segment (2–21 amino acids) to the external face of the inner membrane.

#### 5.3.2. Biochemical Properties

RDH13 prefers NADPH over NADH as its cofactor (20-fold greater) and has retinal reductase activity for all-*trans*-retinal [43].

#### 5.3.3. Animal Studies

No retinal structural and functional abnormalities were found in *Rdh13*^−/−^ mice as compared with WT mice [76]. However, intense light exposure (48 h with 3000 lux) can cause retinal degeneration and attenuation of scotopic ERG. Mitochondrial dependent apoptosis of the retina was also found in *Rdh13*^−/−^ mice [76].

#### 5.3.4. Disease

Although RDH13 has not been associated with any retinal diseases in humans, possible contribution to pathogenesis has been reported in bilateral convergent strabismus with exophthalmus [77].

### 5.4. RDH14

RDH14 was discovered in the retina by Haeseleer et al. [43].

#### 5.4.1. Expression and Localization

Human RDH14 encodes 336 amino acids and locates on chromosome 2 at 2p24.2 and in mice on chromosome 12. Immunoblotting analysis revealed the expression of Rdh14 in bovine cone and rod outer segments and Müller cells [43] (Figure 2). 

#### 5.4.2. Biochemical Properties

RDH14 has shown NADPH dependent catalysis of all-*trans*-retinal, 9-*cis*-retinal, 11-*cis*-retinal and 13-*cis*-retinal isomer. The activity towards 13-*cis*-retinal is lowest among all substrates [43]. RDH14 displays an equal catalysis of 11-*cis*-retinal and all-*trans*-retinal when they are present together in the reaction and exhibit efficient catalysis of reaction in both directions, NAPH/retinals↔NADP^+^/retinols [43]. It has no steroid dehydrogenase activity [43]. The mouse Rdh14 displays retinal-retinol redox (AL-OL)-coupling reaction where it regenerates 11-*cis*-retinal in vitro [14]. Sato et al. identified an efficient regeneration mechanism of 11-*cis*-retinal in carp cone photoreceptors where carp RDH13 and RDH13-like (RDH13L) oxidize 11-*cis*-retinal to 11-*cis*-retinal in presence of hydrophobic aldehydes and does not need exogenous NADPH cofactor [14]. Thus, we speculate that human RDH14 might be involved in aldehyde dependent and NADP^+^ dependent 11-*cis*-retinal production in cone photoreceptor cells.

#### 5.4.3. Animal Studies

No animal studies have been conducted.

#### 5.4.4. Disease 

RDH14 has not been associated with any retinal diseases in humans.

### 5.5. retSDR1/DHRS3 

A conserved domain of retinol dehydrogenase was searched against the EST database and the identified EST clone was confirmed as retSDR1 by Haeseleer et al. in 1998 [78].

#### 5.5.1. Expression and Localization

Human *retSDR1*/*DHRS3* gene locates on chromosome 1 at 1p36.1. retSDR1/DHRS3 expresses predominantly in outer segments of the cone photoreceptors [78] (Figure 2). retSDR1/DHRS3 localizes on the microsomal membrane and anchors to the ER membrane [79].

#### 5.5.2. Biochemical Properties

retSDR1/DHRS3 displays specificity towards all-*trans*-retinal but not 11-*cis*-retinal in the presence of NADPH [78]. Human retSDR1/DHRS3 and RDH10 activate each other in a reciprocal manner to maintain retinoid homeostasis [80]. The activation occurs as a result of protein-protein interaction of retSDR1/DHRS3 and RDH10.

#### 5.5.3. Animal Studies

DHRS3 null mice display postnatal lethality and survive until embryonic stage 18.5 [80,81]. *Ddhrs3*^−/−^ embryo has small eyes, unfused eyelids as compared to WT littermate. *Dhrs3*^−/−^ embryo has also shown sign of edema. *Dhrs3*^−/−^ embryo at Day 13.5 has four-fold-reduced level of retinol and retinyl ester but elevated level of all-*trans*-retinoic acid (atRA). The atRA toxicity is due to increase activity of RDH10 in the early phase of development cause lethality in mice. 

#### 5.5.4. Disease

retSDR1/DHRS3 has not been associated with any retinal diseases in humans. Association between retSDR1/DHRS3 and neuroblastoma has been reported [82].

## 6. Maintenance of Retinoid Homeostasis by RDHs 

Recent discoveries of many dehydrogenases in the RPE and the retina suggest the redundancy of genes for regeneration of visual chromophore. In the RPE, RDH5, RDH11 and RDH10 have been identified to mediate the production of 11-*cis*-retinal. Among these RDHs, RDH5 plays a primary role for this reaction. Similarly, the retina has RDH8, RDH11–RDH14 and retSDR1/DHRS3 for reducing all-*trans* retinal aldehyde to alcohol in the visual cycle. 

In addition to the existence of multiple RDHs, compensatory up-regulation in expression for missing RDHs was observed in mice. *Rdh10* expression was found up-regulated in *Rdh5*^−/−^ in the RPE [32]. Similarly, up-regulation of *Rdh5* gene was detected in RPE-specific *Rdh10* deficient mice. Such up-regulation was evident both in transcriptional and translational levels. This regulation can contribute to maintain the retinoid homeostasis and could be a reason for mild phenotype of *Rdh5*^−/−^ and *Rdh10* cKO mice. 

## 7. Proposed Pharmacologic Treatments for RDH Diseases

### 7.1. Supplementation with 9-cis-Derivatives to Maintain the Visual Cycle

Supplementation with vitamin A derivatives is a potential treatment for retinal diseases that are associated with delayed 11-*cis*-retinal regeneration, such as FA caused by RDH5 mutations. Supplementation of 9-*cis*-retinal to *Rdh5*^−/−^*Rdh11*^−/−^ mice by oral gavage significantly increased rod and cone functions of mice [83]. A pilot clinical trial of supplementation to treat patients with FA was performed. Oral capsules containing 9-*cis*-β-carotene from the alga *Dunaliella bardawil* were administered daily for 90 days. After this treatment, significant increases in the peripheral visual field and rod function measured by electroretinogram were demonstrated [84]. Administration of 9-*cis*-retinyl acetate for a long term to WT mice can increase the visual function in old mice (10 months and 14 months) [85]. This observation suggests a potential benefit of vitamin A supplementation to elder populations who experienced age-related visual dysfunction.

### 7.2. Treatments with Inhibitors to Alleviate from Accumulation of Toxic Visual Cycle By-Products

The visual cycle inhibitors as outlined below debilitate the flux of retinoids in the eye by inhibiting specific steps in the visual cycle. The inhibitors are classified into six groups depending upon their chemical structure and mode of action [86].

#### 7.2.1. Retinoic Acid Derivative

13-*cis*-retinoic acid (13-*cis*-RA, Accutane, Isotretinoin) and hydroxyphenyl amide (4-HPR or fenretinide): 13-*cis*-RA inhibits 11-*cis*-retinol dehydrogenase which is involved in oxidation of 11-*cis*-retinol to 11-*cis*-retinal and decrease the production of chromophore. 11-*cis*-RA also binds to RPE65 to attenuate the 11-*cis*-retinol production [86,87]. Fenretinide reduces the vitamin A/all-*trans*-retinol flux to the eye by interfering with binding of vitamin A to retinol binding protein 4. Retinol binding protein 4 unloads vitamin A cargo in the eye with help of STRA6 receptor [88]. Both 13-*cis*-RA and fenretinide reduce the accumulation of A2E in eye [87,89,90,91].

#### 7.2.2. Positively Charged Retinoids

All-*trans*-retinylamine (Ret-NH_2_) and its derivative: Ret-NH_2_ inhibits 11-*cis*-retinol production by reducing the isomerization of RPR65. It binds to proteins of the RPE microsome but does not bind Rpe65 [92]. Ret-NH_2_ is reversibly converted to retinyl amides by LRAT and stored in the retinosome within the RPE, which slowly hydrolysed to evoke long lasting suppression of retinoid isomerase activity [93] Ret-NH_2_ protects retinal degeneration in *Abca4*^−/−^*Rdh8*^−/−^ mice induce by light exposure [63,86,93]. A non-retinoid derivative of Ret-NH_2_, emixustat (ACU-4429), inhibits isomerarization activity by binding to RPE65 enzyme and protects against light-induced retinal degeneration [94,95]. The production of 11-*cis*-retinol is inhibited more strongly by emixustat as compared to Ret-NH_2_ with prolong blockage of visual pigment regeneration.

#### 7.2.3. Farnesyl-Containing Isoprenoids

These two compounds, (2E,6E)-*N*-hexadecyl-3,7,11-trimethyldodeca-2,6,10-trienamine (TDH) and (12E,16E)-13,17,21-trimethyldocosa-12,16,20-trien-11-one (TDT), limit the production of 11-*cis*-retinal through interaction with RPE65 [96].

#### 7.2.4. Nonretinoid Hydrophobic Primary Amines

Fernesylamine decreases the isomerization reaction and 11-*cis*-retinol production [86].

#### 7.2.5. Primary Amine for atRAL Scavenging

Amine containing compounds such as such as A20S (*S*-3-(aminomethyl)-5-methylhexanoic acid), A20R (*R*-3-(aminomethyl)-5-methylhexanoic acid) and A22 (5-amino-2-hydroxybenzoic acid) make Schiff base with free all-*trans*-aldehyde and reduce the effective concentration of all-*trans*-aldehyde [97]. Primary amine containing compounds protect from light induced retinal degeneration in *Abca4*^−/−^*Rdh8*^−/−^ mice. A20S, A20R and A22 do not cause inhibition of 11-*cis*-retinal production. 

#### 7.2.6. Aromatic Lipophilic Spin Trap Compound

The spin trap compounds, α-phenyl *N*-tertiary-butyl nitrone (PBN), 2-methyl-2-nitrosopropane (MNP), and nitrosobenzene have been identified to trap carotonoids and retinoids radical [98,99]. PBN is found to trap retinyl ester radical by binding to RPE65 and inhibits the isomerohydrolyase activity of RPE65 [100]. The spin trap compounds have great therapeutic potential to modulate the visual cycle in diseases such as Stargardt disease and geographic form of age-related macular degeneration where A2E accumulation is a potential causative agent for vision loss [55,101].

## 8. Conclusions

Continuous regeneration of the visual chromophore through the visual cycle is essential for vision. The reactions of these pathways have been well documented, and RDHs play an important role in this cycle. Further studies of RDHs enzymatic function and regulation in vitamin A metabolism in the eye could lead to development of potential therapeutics for human retinal diseases.

## References

[1] Palczewski K., Verlinde C.L., Haeseleer F. (1999). Molecular Mechanism of Visual Transduction. Novartis Foundation Symposium 224-Rhodopsins and Phototransduction.

[2] Kiser P.D., Golczak M., Maeda A., Palczewski K. (2012). Key enzymes of the retinoid (visual) cycle in vertebrate retina. Biochim. Biophys. Acta Mol. Cell Biol. Lipids.

[3] Muniz A., Villazana-Espinoza E.T., Hatch A.L., Trevino S.G., Allen D.M., Tsin A.T. (2007). A novel cone visual cycle in the cone-dominated retina. Exp. Eye Res..

[4] Matthews R.G., Hubbard R., Brown P.K., Wald G. (1963). Tautomeric Forms of Metarhodopsin. J. Gen. Physiol..

[5] Wald G. (1935). Carotenoids and the Visual Cycle. J. Gen. Physiol..

[6] Wright C.B., Redmond T.M., Nickerson J.M. (2015). A History of the Classical Visual Cycle. Prog. Mol. Biol. Transl. Sci..

[7] Wald G. (1968). The molecular basis of visual excitation. Nature.

[8] Saari J.C. (2012). Vitamin A metabolism in rod and cone visual cycles. Annu. Rev. Nutr..

[9] Johnson P.J., Halpin A., Morizumi T., Prokhorenko V.I., Ernst O.P., Miller R.J. (2015). Local vibrational coherences drive the primary photochemistry of vision. Nat. Chem..

[10] Maeda A., Maeda T., Imanishi Y., Kuksa V., Alekseev A., Bronson J.D., Zhang H., Zhu L., Sun W., Saperstein D.A. (2005). Role of photoreceptor-specific retinol dehydrogenase in the retinoid cycle in vivo. J. Biol. Chem..

[11] Perrault I., Hanein S., Gerber S., Barbet F., Ducroq D., Dollfus H., Hamel C., Dufier J.L., Munnich A., Kaplan J. (2004). Retinal dehydrogenase 12 (RDH12) mutations in leber congenital amaurosis. Am. J. Hum. Genet..

[12] Kaylor J.J., Yuan Q., Cook J., Sarfare S., Makshanoff J., Miu A., Kim A., Kim P., Habib S., Roybal C.N. (2013). Identification of DES1 as a vitamin A isomerase in Muller glial cells of the retina. Nat. Chem. Biol..

[13] Das S.R., Bhardwaj N., Kjeldbye H., Gouras P. (1992). Muller cells of chicken retina synthesize 11-*cis*-retinol. Biochem. J..

[14] Sato S., Miyazono S., Tachibanaki S., Kawamura S. (2015). RDH13L, An enzyme responsible for the aldehyde-alcohol redox coupling reaction (AL-OL coupling reaction) to supply 11-*cis* retinal in the carp cone retinoid cycle. J. Boil. Chem..

[15] Lhor M., Salesse C. (2014). Retinol dehydrogenases: membrane-bound enzymes for the visual function. Biochem. Cell. Biol..

[16] Kavanagh K.L., Jornvall H., Persson B., Oppermann U. (2008). The SDR superfamily: Functional and structural diversity within a family of metabolic and regulatory enzymes. Cell. Mol. Life Sci..

[17] Persson B., Kallberg Y. (2013). Classification and nomenclature of the superfamily of short-chain dehydrogenases/reductases (SDRs). Chem. Biol. Interact..

[18] Wierenga R.K., Terpstra P., Hol W.G. (1986). Prediction of the occurrence of the ADP-binding beta alpha beta-fold in proteins, using an amino acid sequence fingerprint. J. Mol. Biol..

[19] Jornvall H., Persson B., Krook M., Atrian S., Gonzalez-Duarte R., Jeffery J., Ghosh D. (1995). Short-chain dehydrogenases/reductases (SDR). Biochemistry.

[20] Simon A., Hellman U., Wernstedt C., Eriksson U. (1995). The retinal pigment epithelial-specific 11-*cis* retinol dehydrogenase belongs to the family of short chain alcohol dehydrogenases. J. Biol. Chem..

[21] Wang J., Chai X., Eriksson U., Napoli J.L. (1999). Activity of human 11-*cis*-retinol dehydrogenase (*Rdh5*) with steroids and retinoids and expression of its mRNA in extra-ocular human tissue. Biochem. J..

[22] Simon A., Romert A., Gustafson A.L., McCaffery J.M., Eriksson U. (1999). Intracellular localization and membrane topology of 11-*cis* retinol dehydrogenase in the retinal pigment epithelium suggest a compartmentalized synthesis of 11-*cis* retinaldehyde. J. Cell Sci..

[23] Tryggvason K., Romert A., Eriksson U. (2001). Biosynthesis of 9-*cis*-retinoic acid in vivo. The roles of different retinol dehydrogenases and a structure-activity analysis of microsomal retinol dehydrogenases. J. Biol. Chem..

[24] Gonzalez-Fernandez F., Kurz D., Bao Y., Newman S., Conway B.P., Young J.E., Han D.P., Khani S.C. (1999). 11-*cis* retinol dehydrogenase mutations as a major cause of the congenital night-blindness disorder known as fundus albipunctatus. Mol. Vis..

[25] Wada Y., Abe T., Fuse N., Tamai M. (2000). A frequent 1085delC/insGAAG mutation in the RDH5 gene in Japanese patients with fundus albipunctatus. Investig. Ophthalmol. Vis. Sci..

[26] Wang J., Bongianni J.K., Napoli J.L. (2001). The *N*-terminus of retinol dehydrogenase type 1 signals cytosolic orientation in the microsomal membrane. Biochemistry.

[27] Zhang M., Hu P., Napoli J.L. (2004). Elements in the *N*-terminal signaling sequence that determine cytosolic topology of short-chain dehydrogenases/reductases. Studies with retinol dehydrogenase type 1 and *cis*-retinol/androgen dehydrogenase type 1. J. Biol. Chem..

[28] Liden M., Tryggvason K., Eriksson U. (2003). Structure and function of retinol dehydrogenases of the short chain dehydrogenase/reductase family. Mol. Aspects Med..

[29] Driessen C.A., Winkens H.J., Hoffmann K., Kuhlmann L.D., Janssen B.P., Vugt A.H.V., Hooser J.P.V., Wieringa B.E., Deutman A.F., Palczewski K. (2000). Disruption of the 11-*cis*-retinol dehydrogenase gene leads to accumulation of *cis*-retinols and *cis*-retinyl esters. Mol. Cell. Biol..

[30] Jang G.F., Hooser J.P.V., Kuksa V., McBee J.K., He Y.G., Janssen J.J., Driessen C.A., Palczewski K. (2001). Characterization of a dehydrogenase activity responsible for oxidation of 11-*cis*-retinol in the retinal pigment epithelium of mice with a disrupted RDH5 gene. A model for the human hereditary disease fundus albipunctatus. J. Biol. Chem..

[31] Kim T.S., Maeda A., Maeda T., Heinlein C., Kedishvili N., Palczewski K., Nelson P.S. (2005). Delayed dark adaptation in 11-*cis*-retinol dehydrogenase-deficient mice: A role of RDH11 in visual processes in vivo. J. Biol. Chem..

[32] Sahu B., Sun W., Perusek L., Parmar V., Le Y.Z., Griswold M.D., Palczewski K., Maeda A. (2015). Conditional Ablation of Retinol Dehydrogenase 10 in the Retinal Pigmented Epithelium Causes Delayed Dark Adaption in Mice. J. Biol. Chem..

[33] Maeda A., Maeda T., Imanishi Y., Golczak M., Moise A.R., Palczewski K. (2006). Aberrant metabolites in mouse models of congenital blinding diseases: Formation and storage of retinyl esters. Biochemistry.

[34] Yamamoto H., Simon A., Eriksson U., Harris E., Berson E.L., Dryja T.P. (1999). Mutations in the gene encoding 11-*cis* retinol dehydrogenase cause delayed dark adaptation and fundus albipunctatus. Nat. Genet..

[35] Niwa Y., Kondo M., Ueno S., Nakamura M., Terasaki H., Miyake Y. (2005). Cone and rod dysfunction in fundus albipunctatus with RDH5 mutation: An electrophysiological study. Investig. Ophthalmol. Vis. Sci..

[36] Sergouniotis P.I., Sohn E.H., Li Z., McBain V.A., Wright G.A., Moore A.T., Robson A.G., Holder G.E., Webster A.R. (2011). Phenotypic variability in RDH5 retinopathy (Fundus Albipunctatus). Ophthalmology.

[37] Pras E., Pras E., Reznik-Wolf H., Sharon D., Raivech S., Barkana Y., Abu-Horowitz A., Ygal R., Banin E. (2012). Fundus albipunctatus: Novel mutations and phenotypic description of Israeli patients. Mol. Vis..

[38] Wada Y., Abe T., Sato H., Tamai M. (2001). A novel Gly35Ser mutation in the RDH5 gene in a Japanese family with fundus albipunctatus associated with cone dystrophy. Arch. Ophthalmol..

[39] Lin B., White J.T., Ferguson C., Wang S., Vessella R., Bumgarner R., True L.D., Hood L., Nelson P.S. (2001). Prostate short-chain dehydrogenase reductase 1 (PSDR1): A new member of the short-chain steroid dehydrogenase/reductase family highly expressed in normal and neoplastic prostate epithelium. Cancer Res..

[40] Horton J.D., Shimomura I., Brown M.S., Hammer R.E., Goldstein J.L., Shimano H. (1998). Activation of cholesterol synthesis in preference to fatty acid synthesis in liver and adipose tissue of transgenic mice overproducing sterol regulatory element-binding protein-2. J. Clin. Investig..

[41] Kedishvili N.Y., Chumakova O.V., Chetyrkin S.V., Belyaeva O.V., Lapshina E.A., Lin D.W., Matsumura M., Nelson P.S. (2002). Evidence that the human gene for prostate short-chain dehydrogenase/reductase (PSDR1) encodes a novel retinal reductase (RalR1). J. Biol. Chem..

[42] Kasus-Jacobi A., Ou J., Bashmakov Y.K., Shelton J.M., Richardson J.A., Goldstein J.L., Brown M.S. (2003). Characterization of mouse short-chain aldehyde reductase (SCALD), an enzyme regulated by sterol regulatory element-binding proteins. J. Biol. Chem..

[43] Haeseleer F., Jang G.F., Imanishi Y., Driessen C.A., Matsumura M., Nelson P.S., Palczewski K. (2002). Dual-substrate specificity short chain retinol dehydrogenases from the vertebrate retina. J. Biol. Chem..

[44] Kasus-Jacobi A., Ou J., Birch D.G., Locke K.G., Shelton J.M., Richardson J.A., Murphy A.J., Valenzuela D.M., Yancopoulos G.D., Edwards A.O. (2005). Functional characterization of mouse RDH11 as a retinol dehydrogenase involved in dark adaptation in vivo. J. Biol. Chem..

[45] Belyaeva O.V., Stetsenko A.V., Nelson P., Kedishvili N.Y. (2003). Properties of short-chain dehydrogenase/reductase RalR1: Characterization of purified enzyme, its orientation in the microsomal membrane, and distribution in human tissues and cell lines. Biochemistry.

[46] Xie Y.A., Lee W., Cai C., Gambin T., Noupuu K., Sujirakul T., Ayuso C., Jhangiani S., Muzny D., Boerwinkle E. (2014). New syndrome with retinitis pigmentosa is caused by nonsense mutations in retinol dehydrogenase RDH11. Hum. Mol. Genet..

[47] Wu B.X., Chen Y., Chen Y., Fan J., Rohrer B., Crouch R.K., Ma J.X. (2002). Cloning and characterization of a novel all-*trans* retinol short-chain dehydrogenase/reductase from the RPE. Invest. Ophthalmol. Vis. Sci..

[48] Takahashi Y., Moiseyev G., Farjo K., Ma J.X. (2009). Characterization of key residues and membrane association domains in retinol dehydrogenase 10. Biochem. J..

[49] Sandell L.L., Sanderson B.W., Moiseyev G., Johnson T., Mushegian A., Young K., Rey J.P., Ma J.X., Staehling-Hampton K., Trainor P.A. (2007). RDH10 is essential for synthesis of embryonic retinoic acid and is required for limb, craniofacial, and organ development. Genes Dev..

[50] Farjo K.M., Moiseyev G., Takahashi Y., Crouch R.K., Ma J.X. (2009). The 11-*cis*-retinol dehydrogenase activity of RDH10 and its interaction with visual cycle proteins. Investig. Ophthalmol. Vis. Sci..

[51] Bhatia C., Oerum S., Bray J., Kavanagh K.L., Shafqat N., Yue W., Oppermann U. (2015). Towards a systematic analysis of human short-chain dehydrogenases/reductases (SDR): Ligand identification and structure-activity relationships. Chem. Biol. Interact..

[52] Belyaeva O.V., Johnson M.P., Kedishvili N.Y. (2008). Kinetic analysis of human enzyme RDH10 defines the characteristics of a physiologically relevant retinol dehydrogenase. J. Biol. Chem..

[53] Rattner A., Smallwood P.M., Nathans J. (2000). Identification and characterization of all-*trans*-retinol dehydrogenase from photoreceptor outer segments, the visual cycle enzyme that reduces all-*trans*-retinal to all-*trans*-retinol. J. Biol. Chem..

[54] Palczewski K., Jager S., Buczylko J., Crouch R.K., Bredberg D.L., Hofmann K.P., Asson-Batres M.A., Saari J.C. (1994). Rod outer segment retinol dehydrogenase: Substrate specificity and role in phototransduction. Biochemistry.

[55] Sparrow J.R., Parish C.A., Hashimoto M., Nakanishi K. (1999). A2E, a lipofuscin fluorophore, in human retinal pigmented epithelial cells in culture. Investig. Ophthalmol. Vis. Sci..

[56] Birnbach C.D., Jarvelainen M., Possin D.E., Milam A.H. (1994). Histopathology and immunocytochemistry of the neurosensory retina in fundus flavimaculatus. Ophthalmology.

[57] Zhou J., Jang Y.P., Kim S.R., Sparrow J.R. (2006). Complement activation by photooxidation products of A2E, a lipofuscin constituent of the retinal pigment epithelium. Proc. Natl. Acad. Sci. USA.

[58] Ma W., Coon S., Zhao L., Fariss R.N., Wong W.T. (2013). A2E accumulation influences retinal microglial activation and complement regulation. Neurobiol. Aging.

[59] Perusek L., Sahu B., Parmar T., Maeno H., Arai E., Le Y.Z., Subauste C.S., Chen Y., Palczewski K., Maeda A. (2015). Di-retinoid-pyridinium-ethanolamine (A2E) Accumulation and the Maintenance of the Visual Cycle Are Independent of Atg7-mediated Autophagy in the Retinal Pigmented Epithelium. J. Biol. Chem..

[60] Quazi F., Lenevich S., Molday R.S. (2012). ABCA4 is an *N*-retinylidene-phosphatidylethanolamine and phosphatidylethanolamine importer. Nat. Commun..

[61] Maeda A., Maeda T., Golczak M., Palczewski K. (2008). Retinopathy in mice induced by disrupted all-*trans*-retinal clearance. J. Biol. Chem..

[62] Parmar T., Parmar V.M., Arai E., Sahu B., Perusek L., Maeda A. (2016). Acute Stress Responses Are Early Molecular Events of Retinal Degeneration in *Abca4*^−/−^*Rdh8*^−/−^ Mice After Light Exposure. Investig. Ophthalmol. Vis. Sci..

[63] Schur R.M., Sheng L., Sahu B., Yu G., Gao S., Yu X., Maeda A., Palczewski K., Lu Z.R. (2015). Manganese-Enhanced MRI for Preclinical Evaluation of Retinal Degeneration Treatments. Invest. Ophthalmol. Vis. Sci..

[64] Yu Y.S., Wang L.L., Shen Y., Yap M.K., Yip S.P., Han W. (2010). Investigation of the association between all-*trans*-retinol dehydrogenase (RDH8) polymorphisms and high myopia in Chinese. J. Zhejiang Univ. Sci. B.

[65] Kurth I., Thompson D.A., Ruther K., Feathers K.L., Chrispell J.D., Schroth J., McHenry C.L., Schweizer M., Skosyrski S., Gal A. (2007). Targeted disruption of the murine retinal dehydrogenase gene *Rdh12* does not limit visual cycle function. Mol. Cell. Biol..

[66] Maeda A., Maeda T., Imanishi Y., Sun W., Jastrzebska B., Hatala D.A., Winkens H.J., Hofmann K.P., Janssen J.J., Baehr W. (2006). Retinol dehydrogenase (RDH12) protects photoreceptors from light-induced degeneration in mice. J. Biol. Chem..

[67] Belyaeva O.V., Korkina O.V., Stetsenko A.V., Kim T., Nelson P.S., Kedishvili N.Y. (2005). Biochemical properties of purified human retinol dehydrogenase 12 (RDH12): Catalytic efficiency toward retinoids and C9 aldehydes and effects of cellular retinol-binding protein type I (CRBPI) and cellular retinaldehyde-binding protein (CRALBP) on the oxidation and reduction of retinoids. Biochemistry.

[68] Marchette L.D., Thompson D.A., Kravtsova M., Ngansop T.N., Mandal M.N., Kasus-Jacobi A. (2010). Retinol dehydrogenase 12 detoxifies 4-hydroxynonenal in photoreceptor cells. Free Radic. Biol. Med..

[69] Maeda A., Maeda T., Sun W., Zhang H., Baehr W., Palczewski K. (2007). Redundant and unique roles of retinol dehydrogenases in the mouse retina. Proc. Natl. Acad. Sci. USA.

[70] Janecke A.R., Thompson D.A., Utermann G., Becker C., Hubner C.A., Schmid E., McHenry C.L., Nair A.R., Ruschendorf F., Heckenlively J. (2004). Mutations in RDH12 encoding a photoreceptor cell retinol dehydrogenase cause childhood-onset severe retinal dystrophy. Nat. Genet..

[71] Perrault I., Hanein S., Kaplan J. (2004). Leber congenital amaurosis: Retinol dehydrogenases are the culprit. Med. Sci..

[72] Xin W., Xiao X., Li S., Zhang Q. (2016). Late-onset CORD in a patient with RDH12 mutations identified by whole exome sequencing. Ophthalmic Genet..

[73] Weleber R.G., Francis P.J., Trzupek K.M., Beattie C., Pagon R.A., Adam M.P., Ardinger H.H., Wallace S.E., Amemiya A., Bean L.J.H., Bird T.D., Fong C.T., Mefford H.C., Smith R.J.H. (2013). Leber Congenital Amaurosis. GeneReviews^®^.

[74] Hufnagel R.B., Ahmed Z.M., Correa Z.M., Sisk R.A. (2012). Gene therapy for Leber congenital amaurosis: Advances and future directions. Graefe's Arch. Clin. Exp. Ophthalmol..

[75] Belyaeva O.V., Korkina O.V., Stetsenko A.V., Kedishvili N.Y. (2008). Human retinol dehydrogenase 13 (RDH13) is a mitochondrial short-chain dehydrogenase/reductase with a retinaldehyde reductase activity. FEBS J..

[76] Wang H., Cui X., Gu Q., Chen Y., Zhou J., Kuang Y., Wang Z., Xu X. (2012). Retinol dehydrogenase 13 protects the mouse retina from acute light damage. Mol. Vis..

[77] Fink S., Momke S., Distl O. (2012). PLXNC1 and RDH13 associated with bilateral convergent strabismus with exophthalmus in German Brown cattle. Mol. Vis..

[78] Haeseleer F., Huang J., Lebioda L., Saari J.C., Palczewski K. (1998). Molecular characterization of a novel short-chain dehydrogenase/reductase that reduces all-*trans*-retinal. J. Biol. Chem..

[79] Deisenroth C., Itahana Y., Tollini L., Jin A., Zhang Y. (2011). p53-Inducible *DHRS3* is an endoplasmic reticulum protein associated with lipid droplet accumulation. J. Biol. Chem..

[80] Adams M.K., Belyaeva O.V., Wu L., Kedishvili N.Y. (2014). The retinaldehyde reductase activity of *DHRS3* is reciprocally activated by retinol dehydrogenase 10 to control retinoid homeostasis. J. Biol. Chem..

[81] Billings S.E., Pierzchalski K., Tjaden N.E.B., Pang X.Y., Trainor P.A., Kane M.A., Moise A.R. (2013). The retinaldehyde reductase *DHRS3* is essential for preventing the formation of excess retinoic acid during embryonic development. FASEB J..

[82] Kamei N., Hiyama K., Yamaoka H., Kamimatsuse A., Onitake Y., Sueda T., Hiyama E. (2009). Evaluation of genes identified by microarray analysis in favorable neuroblastoma. Pediatr. Surg. Int..

[83] Maeda A., Maeda T., Palczewski K. (2006). Improvement in rod and cone function in mouse model of Fundus albipunctatus after pharmacologic treatment with 9-*cis*-retinal. Investig. Ophthalmol Vis. Sci..

[84] Rotenstreich Y., Harats D., Shaish A., Pras E., Belkin M. (2010). Treatment of a retinal dystrophy, fundus albipunctatus, with oral 9-*cis*-β-carotene. Br. J. Ophthalmol..

[85] Maeda T., Maeda A., Leahy P., Saperstein D.A., Palczewski K. (2009). Effects of long-term administration of 9-*cis*-retinyl acetate on visual function in mice. Investig. Ophthalmol. Vis. Sci..

[86] Golczak M., Maeda A., Bereta G., Maeda T., Kiser P.D., Hunzelmann S., Lintig J.V., Blaner W.S., Palczewski K. (2008). Metabolic basis of visual cycle inhibition by retinoid and nonretinoid compounds in the vertebrate retina. J. Biol. Chem..

[87] Radu R.A., Mata N.L., Nusinowitz S., Liu X., Sieving P.A., Travis G.H. (2003). Treatment with isotretinoin inhibits lipofuscin accumulation in a mouse model of recessive Stargardt’s macular degeneration. Proc. Natl. Acad. Sci. USA.

[88] Kiser P.D., Golczak M., Palczewski K. (2014). Chemistry of the retinoid (visual) cycle. Chem. Rev..

[89] Radu R.A., Han Y., Bui T.V., Nusinowitz S., Bok D., Lichter J., Widder K., Travis G.H., Mata N.L. (2005). Reductions in serum vitamin A arrest accumulation of toxic retinal fluorophores: A potential therapy for treatment of lipofuscin-based retinal diseases. Investig. Ophthalmol. Vis. Sci..

[90] Radu R.A., Mata N.L., Bagla A., Travis G.H. (2004). Light exposure stimulates formation of A2E oxiranes in a mouse model of Stargardt’s macular degeneration. Proc. Natl. Acad. Sci. USA.

[91] Radu R.A., Mata N.L., Nusinowitz S., Liu X., Travis G.H. (2004). Isotretinoin treatment inhibits lipofuscin accumulation in a mouse model of recessive Stargardt’s macular degeneration. Retinal Dystrophies: Functional Genomics to Gene Therapy: Novartis Foundation Symposium 255.

[92] Maeda A., Maeda T., Golczak M., Imanishi Y., Leahy P., Kubota R., Palczewski K. (2006). Effects of potent inhibitors of the retinoid cycle on visual function and photoreceptor protection from light damage in mice. Mol. Pharmacol..

[93] Golczak M., Kuksa V., Maeda T., Moise A.R., Palczewski K. (2005). Positively charged retinoids are potent and selective inhibitors of the *trans*-*cis* isomerization in the retinoid (visual) cycle. Proc. Natl. Acad. Sci. USA.

[94] Kubota R., Boman N.L., David R., Mallikaarjun S., Patil S., Birch D. (2012). Safety and effect on rod function of ACU-4429, A novel small-molecule visual cycle modulator. Retina.

[95] Zhang J., Kiser P.D., Badiee M., Palczewska G., Dong Z., Golczak M., Tochtrop G.P., Palczewski K. (2015). Molecular pharmacodynamics of emixustat in protection against retinal degeneration. J. Clin. Investig..

[96] Gollapalli D.R., Rando R.R. (2004). The specific binding of retinoic acid to RPE65 and approaches to the treatment of macular degeneration. Proc. Natl. Acad. Sci. USA.

[97] Maeda A., Golczak M., Chen Y., Okano K., Kohno H., Shiose S., Ishikawa K., Harte W., Palczewska G., Maeda T. (2012). Primary amines protect against retinal degeneration in mouse models of retinopathies. Nat. Chem. Biol..

[98] Konovalova T.A., Kispert L.D., Polyakov N.E., Leshina T.V. (2000). EPR spin trapping detection of carbon-centered carotenoid and beta-ionone radicals. Free Radic. Biol. Med..

[99] Iwahashi H., Negoro Y., Ikeda A., Kido R. (1987). Detection of some retinoid radicals using high-performance liquid chromatography with electron spin resonance spectroscopy or electrochemical detection. J. Chromatogr..

[100] Poliakov E., Parikh T., Ayele M., Kuo S., Chander P., Gentleman S., Redmond T.M. (2011). Aromatic lipophilic spin traps effectively inhibit RPE65 isomerohydrolase activity. Biochemistry.

[101] Sparrow J.R., Wu Y., Kim C.Y., Zhou J. (2010). Phospholipid meets all-*trans*-retinal: The making of RPE bisretinoids. J. Lipid Res..

